# Folded fabric tunes rock deformation and failure mode in the upper crust

**DOI:** 10.1038/s41598-017-15523-1

**Published:** 2017-11-10

**Authors:** F. Agliardi, M. R. Dobbs, S. Zanchetta, S. Vinciguerra

**Affiliations:** 1 University of Milano-Bicocca, Department of Earth and Environmental Sciences, Piazza della Scienza 4, 20126 Milano, Italy; 2 British Geological Survey, Environmental Science Centre, Nicker Hill, Keyworth, Nottingham, NG12 5GG UK; 3 University of Torino, Department of Earth Sciences, Via Valperga Caluso 35, 10125, Torino, Italy

## Abstract

The micro-mechanisms of brittle failure affect the bulk mechanical behaviour and permeability of crustal rocks. In low-porosity crystalline rocks, these mechanisms are related to mineralogy and fabric anisotropy, while confining pressure, temperature and strain rates regulate the transition from brittle to ductile behaviour. However, the effects of folded anisotropic fabrics, widespread in orogenic settings, on the mechanical behaviour of crustal rocks are largely unknown. Here we explore the deformation and failure behaviour of a representative folded gneiss, by combining the results of triaxial deformation experiments carried out while monitoring microseismicity with microstructural and damage proxies analyses. We show that folded crystalline rocks in upper crustal conditions exhibit dramatic strength heterogeneity and contrasting failure modes at identical confining pressure and room temperature, depending on the geometrical relationships between stress and two different anisotropies associated to the folded rock fabric. These anisotropies modulate the competition among quartz- and mica-dominated microscopic damage processes, resulting in transitional brittle to semi-brittle modes under P and T much lower than expected. This has significant implications on scales relevant to seismicity, energy resources, engineering applications and geohazards.

## Introduction

The interplay between fractures and anisotropic rock fabrics controls the deformation and failure processes of crustal rocks in a variety of tectonic settings^1–4^. The coupled small-scale mechanisms affect the permeability, strength and stiffness of fault zones^5,6^ and crustal rocks^7–10^ at spatial and temporal scales of interest for tectonic deformation and brittle failure^1^, natural and induced seismicity^10,11^, rock reservoirs^12^, and rock engineering^7,8^.

At the microscopic scale, fractures originate from “stress raisers” including pores grain boundaries, weak mineral phases and microcracks^3^. Micro-cracks (several microns to millimetres in length) nucleate and grow stably by subcritical damage propagation, leading eventually to the coalescence into centimetre-scale fractures^2,3,13^. The amount and degree of localization of crack damage accumulated by rock towards failure largely depend on pre-existing cracks and pores, as well as the intrinsic properties of rock texture^3^. However, for the widespread compact rocks (porosity <1.5%^3^), strength and deformation/failure modes are strongly influenced by the fabric anisotropy originated by compositional layering and foliation^4,14–17^.

**Figure 1:**
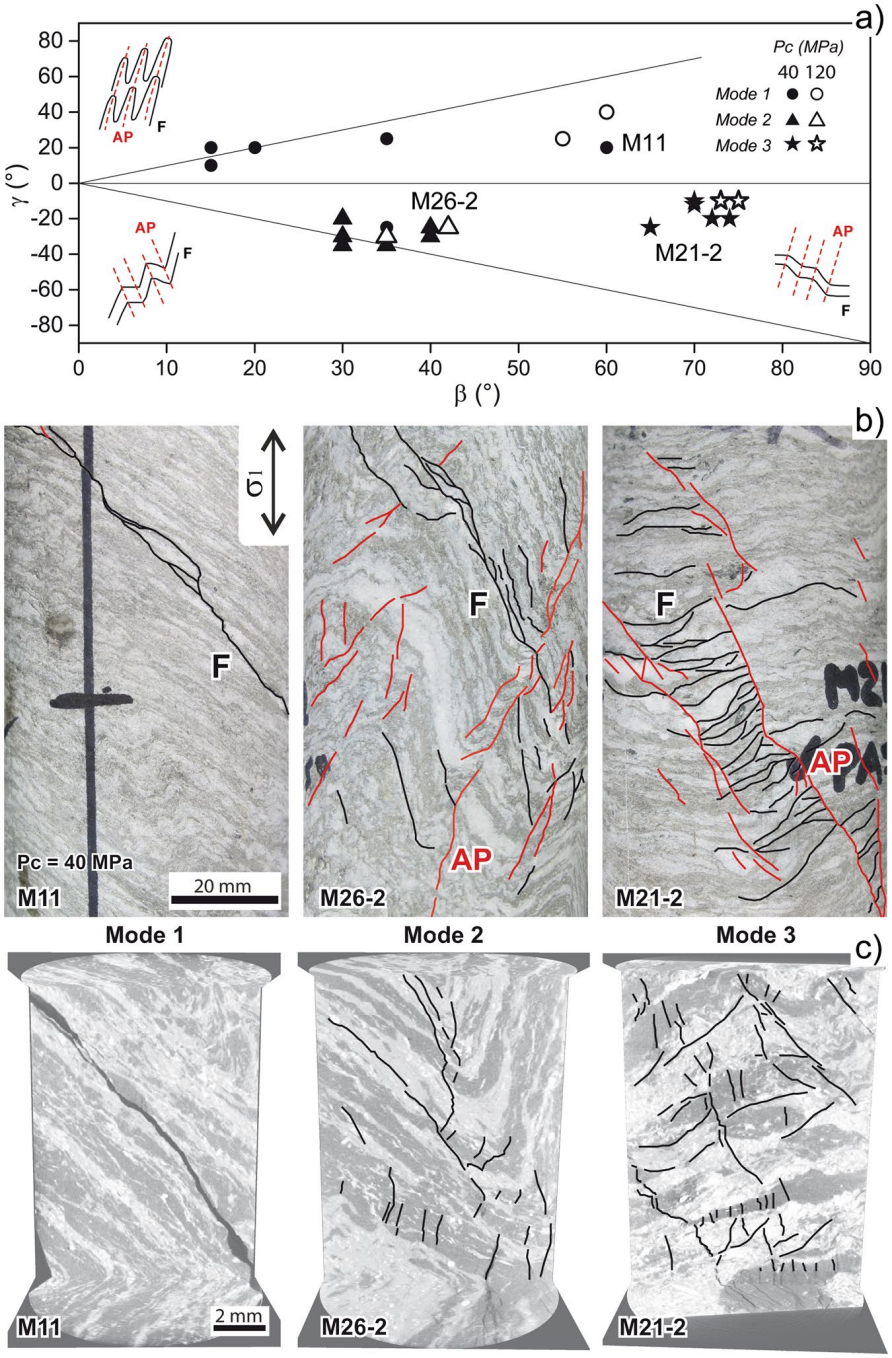
Failure modes of folded gneiss in triaxial compression. (**a**) β-γ plot following^4^ (see Methods) summarizing the geometrical relationships among foliation (β), fold axial planes (γ) and axial load direction corresponding to different failure modes observed in compression experiments (confining P: 40 and 120 MPa). (**b**) fracture maps traced on selected failed specimens (confining P: 40 MPa); (**c**) longitudinal slices (parallel to sample axis) of high-resolution X-ray CT reconstructions (resolution 10 μm, field of view: 9 mm, minimum resolvable crack length: 0.3 mm) with fracture maps typical of different failure modes.

While the influence of planar fabric anisotropy on rock strength has been extensively analysed^3,14–17^, very little is known about the mechanical behaviour of crustal rocks with folded anisotropic fabric, which are widespread ubiquitously and found particularly in collisional tectonic settings, because of the deformation and metamorphic processes that take place there. These rocks are characterized by complex fabric developed at the millimetre- to centimetre-scale, that can be described in terms of fold geometry^18^ and orientation of foliation and fold axial planes to a principal reference direction (e.g. major principal stress, Fig. 1a and Methods). A recent experimental study on the failure mode and rock strength of folded gneiss and schist in uniaxial compression^4^ outlined a primary control of folded fabric on these properties. They systematically depend on the geometrical coupling of two different textural and mechanical anisotropies, namely the foliation and a discrete Axial Planar Anisotropy (APA). The latter arises from microstructural features inherited from ductile tectonic deformation, including narrow bands of quartz subgrains with shape preferred orientation subparallel to fold axial planes, and the occurrence of fractured, mechanically rotated grains, or an embryonic crenulation cleavage in mica-rich domains^4^. However, these results cannot be seen as representative of the rheological behaviour of rocks in upper crustal conditions, as external factors such lithostatic pressure and temperature control the distribution of brittle crack damage^3,19^ as well as the brittle-ductile transition. When brittle microscale failure mechanisms are associated with a macroscopically “ductile” behaviour (i.e. cataclastic flow in the “semi-brittle” regime^19,20^), brittle-ductile transition results in increasing rock strength and deformability and distributed damage^3^. This is expected to change the modes of macroscopic rock fracturing as supported by changes in seismogenic fault slip behaviour near the base of the seismogenic zone^19–21^.

Here we experimentally prove the key control of folded fabric on the mechanisms of fracture initiation and development in crustal rocks at “*in situ*” representative stress conditions, by combining rock deformation laboratory experiments, microseismic output and microstructural observations.

## Rock deformation laboratory experiments

We studied gneiss samples from the Monte Canale tectono-metamorphic unit (Central Alps, Italy). This is a low-porosity (0.8–1.5%) and unaltered granodioritic gneiss made of quartz, K-feldspar, chlorite and white mica, with a total phyllosilicate content lower than 20%^4^ and a bulk rock composition^22^ representative of upper crustal rocks^23^. The texture is characterized by a compositional layering alternating quartz-rich (Q) and mica-rich (M) domains. Structurally, the rock displays tight to gentle harmonic folding at millimetre to centimetre scale, with no axial plane foliation evident at the mesoscale^4^. Outside fold hinge zones, quartz grain size varies between 0.1 and 0.5 mm (modal: 0.2), with only few larger crystals and typical aspect ratio close to 1:1 and up to 1:3 for the larger grains. Mica grains along foliation range in size between 0.3 and 1 mm (modal 0.5 mm). Close to fold hinges (i.e. along axial planes), intracrystalline deformation and recovery due to tectonic processes resulted in the formation of quartz subgrains not larger than few tens of microns, commonly with an aspect ratio of 1:2. At fold hinges, micas show strong intracrystalline deformation with kink bands and fractures, with grain size reduction to about 100 microns where embryonic crenulation foliation occurs.

We performed rock deformation laboratory experiments by carrying out triaxial compression tests on 25 specimens with different orientation of foliation and axial planes (i.e. the characteristic elements of the folded fabric) to the specimen axis (Fig. 1a). The specimens were deformed at constant axial strain rates, in dry conditions, at room temperature and at confining pressures of 40 and 120 MPa (equivalent to about 1.5 and 4.5 km in depth, respectively) while measuring the microseismic output (see Methods) to monitor the evolution of crack damage.

## Folded fabric tunes rock failure modes

**Figure 2:**
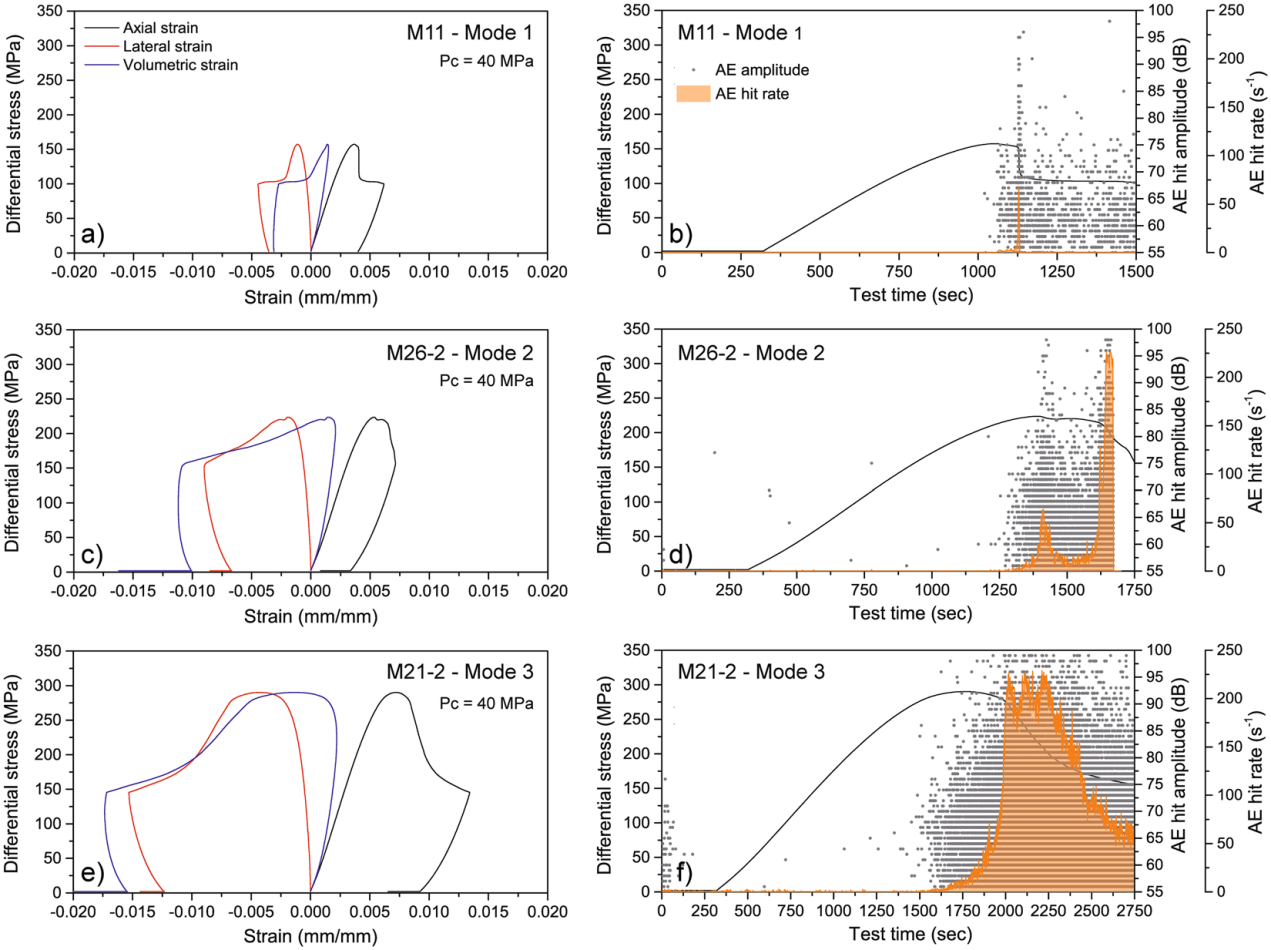
Mechanical and microseismic behaviour of specimens representative of different failure modes. (**a**,**b**,**c**) complete stress-strain curves (axial, lateral and volumetric strains); (**d**,**e**,**f**) curves of differential stress and acoustic emission (AE) hit rate vs time, and amplitudes of AE hits recorded at different test times. Specimens are the same as in Fig. 1.

Our results demonstrate the systematic occurrence of three distinct modes of mesoscopic failure, which correlate with different fold patterns, regardless the fabric complexity (Fig. 1). Failure modes are in good agreement with the results obtained in uniaxial conditions^4^ and were identified at different spatial scales by combining fracture trace maps on sample surfaces (Minimum resolved Crack Length, MCL: 2 mm, Fig. 1b) with cross-section reconstructions of X-ray CT at 10 μm resolution (MCL: 0.3 mm; Fig. 1c) and the analysis of oriented rock thin sections in optical microscopy (MCL: 30–50 μm). Mode 1 failure consists of planar or stepped shear failure along foliation, and is mainly associated with tight to isoclinal folds (Fig. 1). This failure mode is associated with: a) the lowest measured peak strength (<200 MPa); b) a pre-peak accumulated axial strain less than 0.004–0.005 (at 40 and 120 MPa confining pressures, respectively); and c) a sharp post-peak stress drop (Fig. 2a). This corresponds with a typical brittle behaviour^19,20^, as mirrored by the very low acoustic emission (AE) activity. AE starts near the peak of the stress-strain curve and reach a maximum hit rate at the onset of the mesoscopic failure (Fig. 2b). In Mode 2, subsequent shear failure occurs in both M and Q domains along foliation and fold axial planes, i.e. the *loci* of the APA^4^. This failure mode occurs when foliation dips steeply and opposite to axial planes in the sample reference frame. In these conditions, samples exhibit intermediate strength (175–350 MPa depending on confining pressure), an increased pre-peak axial strain (up to 0.005–0.008), and damped and stepped post-peak stress drops (Fig. 2c). Again, AE tracks the failure mechanisms, exhibiting an overall significantly higher number of events, that are more energetic on average and develop more progressively from the onset of dilatancy (Fig. 2d). Finally, Mode 3 is characterised by millimetre-scale brittle shear zones parallel to axial planes, with domino-style rotation of rock *lithons* bounded by the foliation. This mode occurs in samples with gentle folds and foliation gently dipping opposite to axial planes (Fig. 1). Mode 3 failure is always associated with the highest strength values (up to 300–450 MPa depending on confining pressure), very large pre-peak axial strain (up to 0.007–0.013), and damped stress drop (Fig. 2e). These behaviours indicate a transition from brittle to semi-brittle^19,20,24^ supported by abundant AE activity from the onset of dilatancy. AE hit rates and event amplitudes increase progressively until mesoscopic sample failure is reached (Fig. 2f), consistently with extensive pre-peak distributed cracking^25,26^. At confining pressure of 120 MPa, we recognised identical failure modes and geometrical relationships with folded fabric, but more distributed mesoscopic failures, consistent with the confining pressure influence on the brittle-ductile transition. Indeed, evidence of this transition occurs at lower confining pressure than in non-folded rocks with comparable mineralogy^3,24^.

Our experimental dataset shows that, in upper crustal stress conditions, folded gneiss is weaker than similar rock types with a planar fabric, and fails in a much more complex fashion. Gottschalk *et al*.^17^ reported that the Four mile gneiss, compositionally similar to the Monte Canale gneiss, failed under triaxial compression by conjugate brittle fractures with different degree of symmetry depending on the orientation and spatial arrangement of mica grains^17^. In addition, the Four mile gneiss exhibited a classical continuous strength anisotropy^3^, with peak differential stress at failure varying with the orientation of the main foliation with respect to the differential stress direction^17^. Instead, the failure modes of folded gneiss and its strength are strongly heterogeneous and systematically correlated with fold geometry and orientation (β-γ plot in Fig. 1a). Failure modes involving the APA (modes 2 and 3) show mechanical and microseismic (AE) evidence of transitional behaviour from purely brittle to semi-brittle, including increasing strength, deformability and damage delocalization by distributed microcracking. AE is two orders of magnitude more abundant in failure mode 3 (involving both foliation and the APA) that in mode 1 (involving foliation only). Moreover, in mode 3 there is a more distinct increase of high-amplitude events, consistent with a significant progressive crack damage accumulation preceding failure through quartz-rich (Q) domains.

## Quartz- vs. mica-dominated microscopic damage processes

**Figure 3:**
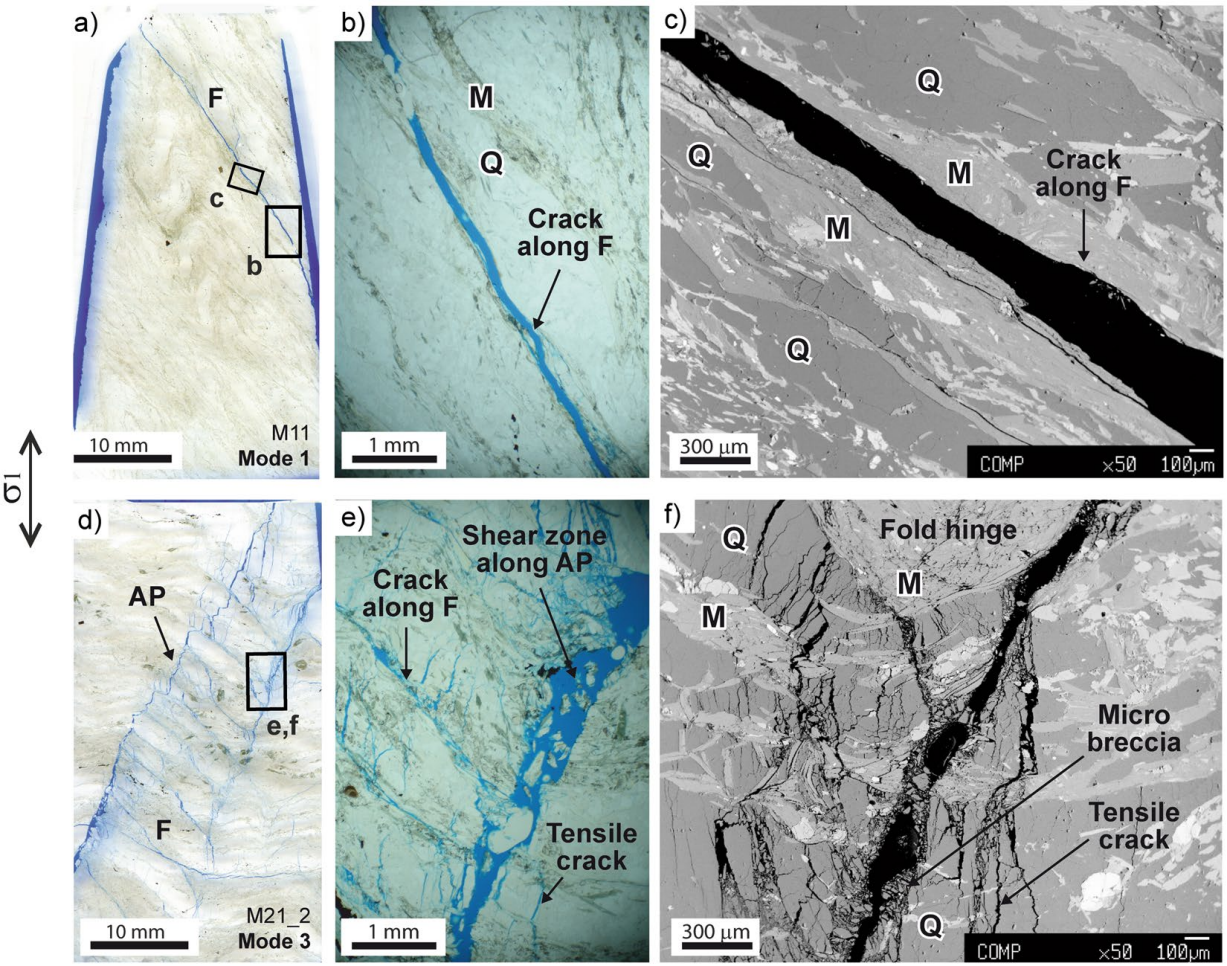
Microscopic evidence of brittle damage associated with different failure modes. Images are from optical and SEM microscopy on oriented thin sections, cut perpendicular to the main failure planes or shear zones (see Methods). (**a**,**b**,**c**) optical and SEM images of neat shear failure along foliation inside M-domains (failure mode 1: brittle, “mica-dominated” damage pattern); (**d**,**e**,**f**) optical and SEM images of complex brittle fracture process zones parallel to the Axial Plane Anisotropy and cutting through multiple Q-domains and M-domains (failure mode 3: transitional to semi-brittle, “quartz-dominated” damage pattern).

We investigated the microscale cracking mechanisms underlying macroscopic failure modes by standard optical microscopy and SEM imaging on oriented rock thin sections (minimum resolvable crack length about 5–10 μm at 50x magnification; see Fig. 3). These observations confirm that a prominent shear failure within sub-millimetric M domains or along M-Q domain boundaries underlies Mode 1 failure (Fig. 3a,b). Very few microcracks form around the main fracture zones in M domains, and very limited comminution of phyllosilicate grains occurs along the fracture rims (Fig. 3c). These observations are consistent with the very limited pre-peak AE activity (negligible crack damage before failure). On the opposite side, Mode 3 failure shows evidence of complex crack damage patterns associated with failure along the APA (Fig. 3d,e). Failure in compression nucleates in Q domains as trans-granular tensile cracks subparallel to the major principal stress direction, still evident in the broken sample away from the main fracture zones (Fig. 3f). Trans-granular cracks interact with a network of microcracks, branching within quartz subgrain aggregates along the APA^4^ and promoting the development of millimetre-scale shear fractures along axial planes favourably oriented to the differential stress σ_1_ − σ_3_ (Fig. 3f). These ones are connected by shear fractures along foliation, bounding micro-lithons with high microcrack density completing the microstructure of brittle shear zones typical of Mode 3 (Fig. 3d,e). Evidence of comminution and micro-breccia/gouge production along shear fractures outline the importance of cataclastic processes in Mode 3 failure. Microscale observations explain the abundance, trend and amplitude of AE in Mode 3 failure. Mode 2 failure involves a combination of failure along foliation and across Q domains at the microscale, and thus exhibits an intermediate mechanical behaviour.

**Figure 4:**
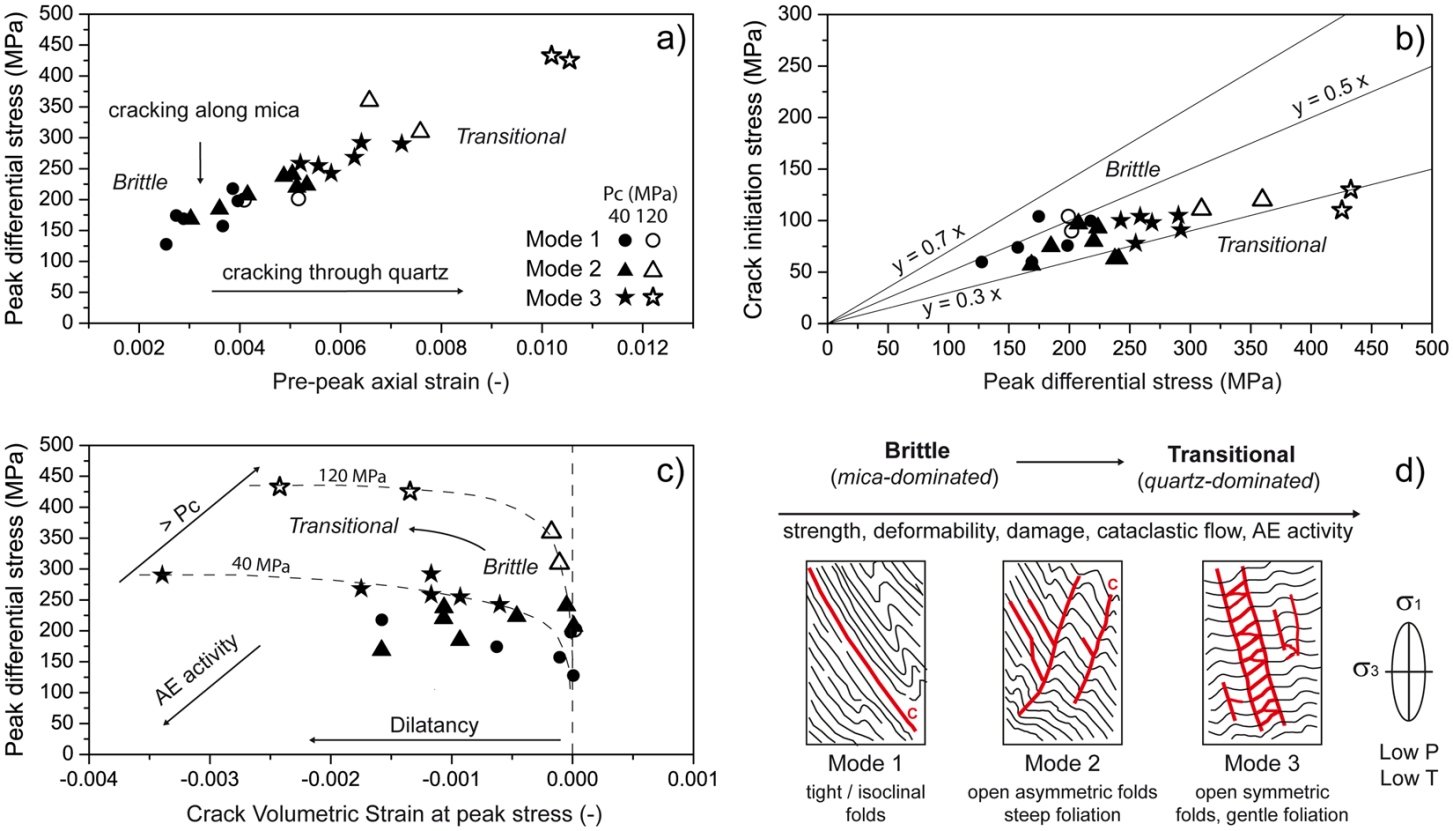
Conceptual model constrained by damage proxies (complete experimental dataset). (**a**) peak differential stress (PDS) vs. pre-peak axial strain shows clear separation among different failure modes (both at 40 and 120 MPa confining pressure); (**b**) crack initiation stress vs. PDS highlights the departure from brittle to a transitional behaviour for rock failed in “quartz-dominated” modes (2 and 3); (**c**) PDS vs. Crack Volumetric Strain shows higher dilatancy and strength associated to transitional behaviour in “quartz-dominated” failure modes, whereas rocks failed in “mica-dominated” mode 1 are weaker and brittle. (**d**) conceptual model of the observed mechanical behavior.

Mechanical proxies further highlight the evolution of the microscopic brittle damage^13,27–29^ (Fig. 4, see Methods), integrating our experimental and microstructural observations and allowing to develop a conceptual model of the mechanical behaviour observed. At constant confining pressure, fabric-stress geometrical coupling conditions promoting failure along foliation (failure mode 1) are associated with the lowest strength values and pre-peak axial strain (Fig. 4a). In these conditions, the rock starts cracking at stress levels comparable to the majority of brittle rocks (45–55% of peak stress^13,28^, Fig. 4b) with very low Crack Volumetric Strain^27,30^ (CVS, i.e. a well-established proxy of crack-related dilatancy, see Methods) at peak stress, indicating very limited crack damage before sample failure (Fig. 4c). Conversely, in conditions increasingly involving failure along the APA through M and Q domains (failure modes 2 and 3), a linear increase in strength and pre-peak axial strain is observed (Fig. 4a). Microcrack growth across Q domains starts at much lower stress levels than reported for brittle low-porosity rocks^28^ (Fig. 4b). Distributed crack damage accumulation and comminution among and within small quartz grains along the APA occur over a wide stress range before peak stress (Fig. 4b). Here the rock fails in a less localized mode than simply along foliation, attesting a clear switch to a transitional mechanical behaviour (Fig. 4d). Our observations also agree with other experimental studies^13,29^, showing that microcracking through quartz aggregates involves higher fracture energy, frictional mobilization and strain than through phyllosilicate aggregates. Different mechanisms related to microscale fabric heterogeneity and anisotropy may interplay to contribute to this behaviour, including microcrack deflection at interfaces between grains or fabric domains with different toughness^31,32^. Experiments carried out at higher confining pressures (120 MPa) reflect the same behaviour described above, with an enhanced tendency towards a semi-brittle behaviour (Fig. 4).

## Discussion

Our results demonstrate that the mechanical response to stress of typical folded gneiss is controlled by the competition among mica- and quartz-dominated microscale damage and failure mechanisms, resulting in contrasting brittle failure modes and transitional behaviours (Fig. 4d). Thus, in stress conditions of the upper crust, the same folded rocks can exhibit contrasting mechanical behaviours at identical lithostatic pressure and room temperature, simply by modifying the geometrical relationships between two fold-related textural anisotropies (i.e. foliation and APA) and the boundary stress state. When these relationships favour “quartz-dominated” microscopic brittle failure processes, the rock undergoes an initial transition from brittle to semi-brittle behaviour (cataclastic flow), at P and T conditions under which only brittle behaviour is expected in quartz-bearing rocks. Therefore, folded fabric modulates the brittle-ductile transition of upper crustal rocks, prevailing on the effects of pressure and temperature. Our findings highlight the importance of characterizing complex geometries such ones arising from the overlap of foliations and folds when such rocks are investigated. The interaction among the above mentioned structures carries main implications for the study of the seismic/aseismic fault slip behaviour (i.e. slow-slip vs. stick-slip at shallow depth^33^), the evaluation of damage-dependent fluid transport properties of the upper crust, and the engineering performance of folded rocks in underground projects or geo-energy exploitation operations (e.g. crack propagation in hydrofracturing).

## Methods

### Sample material preparation and characterization

The mineral composition, petrography and fabric of the Monte Canale gneiss (from now on MCG) had been previously characterised using quantitative X-Ray powder diffraction (XRPD), optical microscopy on differently oriented thin sections (i.e. perpendicular to foliation, fold axes and axial planes, or parallel to axial planes and fold axes), and SEM imaging of carbon-coated raw rock sample surfaces^4^. In this study we tested 25 cylindrical specimens of MCG with a diameter of 54 mm, height between 110 and 130 mm, and aspect ratio (i.e. cylinder height/diameter) between 2.2–2.4. We obtained the specimens by undercoring drillcore segments with a diameter of 78 mm, using a waterfed diamond core bit mounted on a pillar drill. Specimens were trimmed and ground to meet standard flatness and perpendicularity requirements^34,35^. For each specimen, we determined the density and effective porosity using standard bulk volume saturation/buoyancy techniques^34^. We also characterized the geometrical features of the folded meso-fabric^4^ using representative values of fold amplitude (A), wavelength (λ), and inter-limb angle (α), according to widely used fold terminology^18^ and assuming cylindrical folds. We combined these parameters to obtain a quantitative description of the geometrical relationships between foliation (main textural source of anisotropy), axial planes of cm-scale folds (the Axial Plane Anisotropy, a non-pervasive anisotropy related to inherited deformation and recovery micro-structures at fold hinge zones^4^), and specimen longitudinal axis (also the direction of axial compressive loading). These relationships are quantified by the angles β (angle between dip line of the foliation and specimen’s longitudinal axis) and γ (angle between dip line of fold axial planes and specimen’s longitudinal axis), and represented in a β-γ plot (Fig. 1a). Specimens were selected in order to obtain as many β-γ combinations as was possible from the drillcore.

### Rock deformation experimental set up

We performed the triaxial deformation experiments at the BGS Rock Mechanics and Physics laboratory (RMPL) using a large-scale rock deformation apparatus, able to measure the physical properties and behaviour of rocks at pressure and temperature conditions associated with near-surface to shallow crustal environments (about 6 km). The system comprises a 4.6 MN stiff servo-hydraulic load frame (MTS 815), a deviatoric-type pressure vessel and confining pressure intensifier unit (cell pressure up to 140 MPa). Specimens for triaxial tests are encased in heat shrink PTFE and instrumented with direct contact strain gauges (two axial and one circumferential). The setup allows synchronous measurements of axial load, confining pressure, axial strain (ε_ax_) and circumferential strain (ε_cir_). Volumetric strain was calculated using the relation ε_vol_ = ε_ax_ + 2ε_cir_ (Fig. 2a,b,c). We carried out triaxial deformation experiments at confining pressures of 40 MPa (19 experiments) and 120 MPa (6 experiments), to investigate the influence of different crustal stress conditions on the mechanical behaviour of MCG and the related fabric controls. Experiments were conducted in dry conditions at 20 °C using a custom procedure with the following steps: a) 5 kN axial preload; b) cell pressure increase to 40 MPa or 120 MPa in 4–5 minutes while maintaining constant axial preload; c) deviatoric (axial) loading at a servo-controlled strain rate of 5 × 10^–6^ s^−1^, up to the attainment of peak-stress; d) further axial strain accumulation up to 1% post peak-stress; e) unloading at 2.3 kN/s to preload conditions; f) cell pressure unloading while maintaining constant axial load. This experimental setup and procedure captured the complete stress-strain curves of the tested specimens^36^ (Fig. 2a,b,c). We measured the energy radiated by specimens in the form of Acoustic Emissions (AE) for 11 selected experiments using an MTS ultrasonic velocity platen housing three wideband (350–1000 kHz) piezoelectric transceivers (two S-wave, orthogonally polarized, and one P-wave) connected to three 40 dB (100–1200 kHz) preamplifiers. Acoustic data were acquired with a Physical Acoustics (PAC) 16-bit PCI-8 digital signal processing board and processed by the software PAC AEwinRockTest^TM^. AE were recorded in a triggered mode using a threshold of 55 dB. AE hits were defined according to a set of timing parameters (rise time out, hit definition time, rearm time out) and individually characterized in terms of rise time, amplitude, average frequency, duration, and radiated energy. AE data were synchronized with the test time and are presented in terms of hit number and hit rate versus time (Fig. 2c,d,e).

### Post-failure analysis

Our post-failure analysis of specimens deformed in the triaxial experiments included: (a) observation and mapping of sample-scale fracture patterns to identify typical failure modes (Fig. 1b); (b) microscale observations of damage and failure mechanisms underlying the three different observed failure modes (Fig. 3); and c) the associated mechanical and micro-seismic behaviour (Figs 2 and 4). We initially kept post-failure specimens in their PTFE jackets in order to preserve the mesoscopic failure pattern. Specimens not affected by excessive deformation associated to dynamic failure (which occurred for 6 specimens at 40 MPa confining pressure) were removed from jackets, impregnated with blue-dye epoxy resin, and cut perpendicular to the mesoscopic failure planes or shear zones to produce standard optical thin sections. We also made mesoscale observations of the surface of post-failure specimens (Fig. 1b), and the inside of post-failure specimens using X-ray Computed Tomography. X-ray CT imaging allowed us to characterize: a) the 3D pattern of brittle fractures produced by the triaxial compression experiments, and b) the relationship between brittle fractures and rock fabric^4,37^. We scanned the post-failure specimens at different resolution levels (i.e. voxel size) using the BIR Actis 130/150 MicroCT/DR system hosted at the UNIMIB Rock Mechanics laboratory. Whole specimens were scanned at a 55 μm voxel size, whereas remnant rock slices from thin section production were used for very fine resolution scans (voxel: 10 μm) of the most significant failure patterns (Fig. 1c). We analysed CT image stacks using ImageJ^38^ and Avizo Fire (FEI-VSG^TM^) software to improve the description of the three observed mesoscopic failure modes (Fig. 1c) and their relationships with rock fabric (β-γ plot). Fractures were identified by segmenting CT image stacks with calibrated binary thresholds without noise filtering. At 10 μm scan resolution, we were able to resolve a (conservative) minimum crack length value of about 0.3 mm in the CT imaging (Fig. 1c). Our microscale observations were made using standard optical microscopy on crack patterns enhanced by the blue-dye and using a JEOL 8200 scanning probe with accelerating voltage of 15 kV in backscattered electron (SEM-BSE) mode. Minimum resolved crack length values obtained with optical microscopy (30–50 μm) and SEM imaging (5–10 μm at 50x magnification, depending on crack orientation) are small enough to allow analysis of crack patterns in brittle low-porosity rocks^30^.

### Diagnostic criteria for brittle-semibrittle transition

We discriminated between brittle and ductile mechanical behaviours based on a review of the most established and objective criteria available in the literature for low-porosity rocks^3,39^. These include damage proxies based on stress-strain curves, microscopic failure evidence and their acoustic signatures. While the term “brittle” is associated with distinctive features of marked strain softening and strain localization at both macro- and microscales, in rock mechanics the term “ductile” is a rheological one, which refers to the capacity for substantial shape change without gross fracturing and localization on the macroscopic scale^3^. This can be the result of substantially different micro-scale mechanisms of strain accumulation and failure, including cataclastic flow, crystal plasticity or diffusional flow depending on confining pressure, temperature conditions, rock composition, and scale of consideration. This results in the definition of a “semi-brittle” behaviour^19,20^, i.e. a macroscopically ductile behaviour characterised by the dominance of cataclastic flow (i.e. combination of microcracking and frictional sliding^3^) on the microscopic scale. The occurrence of cataclastic flow in rocks with negligible initial porosity at low temperature (i.e. shallow crustal level) is mirrored by dilatancy and acoustic emission activity^3,21,26^. At room temperature, the transition from a brittle to a semi-brittle behavior commonly occurs at confining pressures exceeding 300 MPa for schistose rocks and 600 MPa for quartz-bearing rocks^3^. Typical features of this transition include^21^:

- increasing strength and “damped” post-peak stress drop^21,40^;
- high accumulated axial strain at peak stress (Fig. 4a), with values of about 3–5% at high confining pressure sometimes adopted to define the transition^21,26,41^;
- progressive delocalization of mesoscopic shear zones, accompanied by increasingly distributed microcracking over a wide stress range^3^;
- no crystal plasticity in quartz, but pressure-dependent strength and dilatancy associated with intense cracking within, and between, quartz grains, accompanied by grain crushing, comminution and gouge formation in micro-shears along grain boundaries^3,21^.

### Proxies of microscopic brittle damage

In order to discriminate brittle and transitional behaviour, and to correlate them with the mesoscopic failure modes (Fig. 1) observed in our experiments, we used three proxies of microscopic damage derived from analysis of our experimental stress-strain curves (Fig. 2):

- the axial strain accumulated at peak stress (Figs 2a,b,c and 4a) is a proxy of the amount of distributed damage in the specimen, with higher values associated with transition to semi-brittle behaviour^21,26^, and is correlated with the corresponding microscopic failure mechanisms (Fig. 3) and their folded fabric controls^4^;
- the crack initiation stress (Ci) is the stress level at which the stress-volumetric strain curve deviates from linearity (i.e. elastic stage) due to the subcritical nucleation of microcracks and related “onset of dilatancy”. The ratio of crack initiation stress to peak stress (Fig. 4b) is mostly in the range 0.45–0.55 for brittle rocks^42^ and decreases in the transition to semi-brittle. To account for the uncertainty affecting the evaluation of crack initiation stress from stress-strain curves, we compared the results of different methods^27,42^;
- the Crack Volumetric Strain (CVS^42^) is a measure of inelastic strain derived by subtracting the elastic volumetric strain (ε_v_)_E_ from the total experimental volumetric strain (ε_v_), with (ε_v_)_E_ = (σ_1_ − σ_3_)(1 − 2*v*)/E^27,30^, where v and E are the Poisson ratio and Young modulus, respectively, experimentally derived from the linear portion of the stress-strain curve. For each triaxial experiment we derived the curves of CVS according to Katz and Reches^30^. For each loading step, the CVS reflects microcrack opening mirrored by dilatancy, and its value at peak stress (Fig. 4c) provides a measure of the crack damage accumulated at failure.

These proxies were combined with the meso- and micro-scale observations of failure patterns, and AE data, to investigate the relationships between microscopic damage patterns and mechanisms, mesoscopic failure modes, their fabric controls, and brittle to semi-brittle transition.

### Data availability

The datasets generated and analysed during the current study are available in raw or table format from the corresponding author on reasonable request.
